# Relationship Between Early Cartilage Degeneration With T1ρ and T2 Analysis in Medial Tibial Cartilage and 10-Year Posttraumatic Osteoarthritis Progression After ACL Reconstruction

**DOI:** 10.1177/03635465251360800

**Published:** 2025-09-01

**Authors:** Shotaro Watanabe, Takuma Kaibara, C. Benjamin Ma, Virginie Kreutzinger, Katharina Ziegeler, Rupsa Bhattacharjee, Brian T. Feeley, Thomas M. Link, Sharmila Majumdar, Drew A. Lansdown

**Affiliations:** * Department of Orthopedic Surgery, Sports Medicine & Shoulder Surgery, University of California, San Francisco, San Francisco, California, USA; † Department of Orthopaedic Surgery, Graduate School of Medical and Pharmaceutical Sciences, Chiba University, Center for Preventive Medical Sciences, Chiba University, Chiba, Japan; ‡ Department of Orthopedic Surgery, Hokkaido University Graduate School of Medicine, Sapporo, Japan; § Department of Radiology and Biomedical Imaging, University of California, San Francisco, San Francisco, California, USA; Investigation performed at University of California, San Francisco, San Francisco, California, USA

**Keywords:** anterior cruciate ligament reconstruction, T1ρ/T2 mapping, posttraumatic osteoarthritis, quantitative MRI

## Abstract

**Background:**

Quantitative magnetic resonance imaging (qMRI), such as T1ρ and T2 mapping, allows for the characterization of the biochemical composition of cartilage and the detection of early degeneration within 1 year after anterior cruciate ligament reconstruction (ACLR). It remains unclear if early imaging biomarkers are associated with the long-term development of posttraumatic osteoarthritis (PTOA).

**Hypothesis/Purpose:**

The study aimed to investigate the relationship between qMRI-based evaluation of the medial compartment at baseline and 1 year after ACLR and the knee joint morphological status using 3-dimensional (3D) MRI at a 10-year follow-up. It was hypothesized that elevated T1p and T2 values at 1 year after ACLR would correlate with increased degenerative joint disease at 10 years after ACLR.

**Study Design:**

Cohort study; Level of evidence, 3.

**Methods:**

Patients with ACLR between 2011 and 2014 who had preoperative bilateral qMRI were included. Thirteen patients completed the 10-year follow-up and were evaluated. The mean T1ρ and T2 values at baseline and 1 year were obtained using in-house–developed software with MATLAB. The MRI scans at the 10-year follow-up were acquired using a 3-T MRI scanner, including bilateral 3D proton-density fat-saturated fast-spin-echo sequence. Two radiologists used the modified Whole-Organ Magnetic Resonance Imaging Score (WORMS) to grade the images. The correlations between baseline, 1-year, and delta (1 year – baseline) T1ρ/T2 values and the WORMS at 10 years postoperatively were analyzed using the Spearman correlation coefficient. Significance was defined as a *P* value <.05.

**Results:**

The baseline, 1-year, and delta medial tibia (MT) T1ρ values were significantly associated with the 10-year WORMS Total Score (*r* = 0.604, *P* = .031; *r* = 0.604, *P* = .031; and *r* = 0.582, *P* = .039, respectively). The MT T2 value at 1 year postoperatively was significantly associated (*r* = 0.566; *P* = .046) with the 10-year WORMS Total Score.

**Conclusion:**

The T1ρ/T2 values 1 year postoperatively and the increase from baseline to 1 year in the MT cartilage after ACLR were associated with the deterioration of the comprehensive evaluation of the knee at 10 years postoperatively. These findings help establish qMRI as a valuable biomarker for detecting early signs of PTOA progression.

Anterior cruciate ligament (ACL) injuries are frequently seen injuries in active and young populations. After ACL injury, there is an inflammatory cascade, along with persistent alterations in tibiofemoral kinematics, that results in an increased risk of developing early posttraumatic osteoarthritis (PTOA). There is currently no treatment that can reverse the progression of PTOA. One of the challenges in studying potential interventions to limit the onset and progression of PTOA after ACL injury is the significant lag time from injury and potential intervention to the development of the condition. Symptomatic and radiographic PTOA develops >10–15 years after the initial ACL reconstruction (ACLR).

Quantitative magnetic resonance imaging (qMRI), such as T1ρ and T2 mapping, was developed to detect qualitative changes in the biochemical composition of tissues. The T1ρ relaxation time correlates with the content of proteoglycans, and the T2 relaxation time correlates with the collagen structure of tissues.^14,22^ Quantitative MRI has been suggested as a novel method for detecting and tracking early signs of cartilage degeneration.^3,36^ Quantitative MRI has been utilized to evaluate patients after ACLR, demonstrating detectable differences between injured and uninjured knees before and after ACLR.^5-7,9,27,28,30,31,33^ Numerous studies have reported increases in T1ρ and T2 values in the cartilage of the medial compartment during the early postoperative period.^1,16,27,30^ It remains unclear if these early observations are correlated with the eventual development of PTOA.

Therefore, the present study aimed to investigate the relationship between the qMRI values in the medial compartment early after ACLR and the knee joint morphological status using 3-dimensional (3D) MRI at a 10-year follow-up. We hypothesized that early postoperative increases in T1ρ and T2 values in the medial compartment were associated with increased evidence of PTOA measured by WORMS 10 years postoperatively.

## Methods

### Participants

A total of 13 patients (9 male, 4 female) with a mean age of 32.4 ± 5.4 years at surgery were included and evaluated. Patients were included from 2 previous prospectively recruited longitudinal cohort studies.^1,2,7,8,13,21,24,28,35^ These previous studies included 76 patients with traumatic unilateral ACL injuries between July 2011 and September 2014. At the enrollment of the previous cohort studies, patients who were unable or unwilling to consent to the present study, had a history of previous knee trauma, had previous knee surgery, required cartilage repair, had a joint inflammatory disease, or had diagnosed osteoarthritis (OA) with Kellgren-Lawrence grade >1 on the preoperative radiograph were excluded. For inclusion in the current study, patients were included if they had preoperative (baseline) bilateral knee MRI and 1-year postoperative MRI scans available and were willing to return for follow-up imaging. All procedures were approved by our institutional review board, and all patients provided documented informed consent.

### Surgical Procedure and Rehabilitation

All procedures were performed by 1 of 3 board-certified, fellowship-trained orthopaedic surgeons (including C.B.M. and B.T.F.). All patients underwent single-bundle ACLR using a soft tissue graft, with 8 receiving hamstring tendon autografts and 5 receiving posterior tibialis allografts. The standard postoperative rehabilitation protocol, as previously described,^25^ was followed. Tunnels were drilled independently, and fixation was achieved with suspensory fixation on the femoral side with interference screw fixation at the tibial tunnel.

### Quantitative MRI Acquisition at Baseline and 1 Year Postoperatively

**Figure 1.**
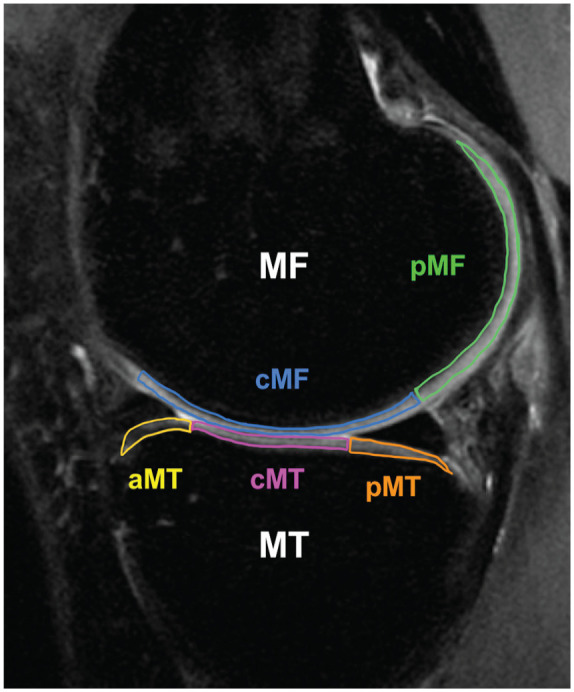
Segmentation of the sagittal magnetic resonance imaging scan of the medial compartment. The medial femur (MF) and medial tibia (MT) are divided into subcompartments with respect to the meniscus. aMT, anterior MT; cMF, central medial femur; cMT, central MT; pMF, posterior medial femur; pMT, posterior MT.

The MRI scans at baseline and 1 year after surgery were acquired using a 3-T MRI scanner (GE HealthCare) with an 8-channel knee coil (Invivo). The imaging protocol included sagittal high-resolution 3D fast-spin-echo (FSE) images (TR/TE, 1500/25 ms; echo train length, 32; matrix, 384 × 384 pixels; field of view [FOV], 16 cm; slice thickness, 1 mm [interpolated into 0.5 mm]) to evaluate cartilage and meniscal morphology, and a sagittal T1ρ/T2 quantification sequence (TR/TE, 8/3 ms; FOV, 14 cm; matrix, 256 × 128 pixels; slice thickness, 4 mm; views per segment, 64; spin-lock frequency, 500 Hz; T1ρ time of spin-lock: 0, 10, 40, and 80 ms; T2 preparation TE, 0, 13.7, 27.3, and 54.7 ms for 8 cases, 0, 12.8, 25.7, and 51.4 ms for 5 cases). The high-resolution 3D FSE images were downsampled in the sagittal direction and registered to the first echo of the T1ρ/T2 sequence as previously described.^18^ The postprocessing for T1ρ and T2 analysis was performed using an in-house program developed in MATLAB (Version R2021a; The MathWorks) integrated with the Elastix library for image registration.^12,23^ Cartilage was segmented semiautomatically on 3D FSE images using an algorithm based on edge detection and Bezier splines.^4^ We performed semiautomatic cartilage segmentation on the 3 or 4 slices in which that region was visible. We calculated the mean values across these 3 or 4 slices in each cartilage region of interest: medial femur (MF), medial tibia (MT), lateral femur (LF), and lateral tibia (LT). Because previous research has suggested early cartilage degeneration in the medial compartment after ACLR,^1,16,27,30^ the MF was further subdivided into 2 subcompartments based on the meniscus: central MF and posterior MF (pMF). Similarly, the MT was also further subdivided into 3 subcompartments concerning the meniscus: anterior MT, central MT, and posterior MT (pMT) (Figure 1). The color map image was reviewed and manually adjusted to exclude the surrounding water from the segmentation. The difference in values from baseline to 1 year (delta T1ρ and delta T2) was calculated for each region and subcompartment.

### Ten-Year Follow-up After ACLR

#### Modified Whole-Organ Magnetic Resonance Imaging Score

All MRI scans 10 years after surgery were acquired bilaterally using a 3-T GE Signa Premier scanner (GE HealthCare) with two 16-channel medium flex receive-only coils (NeoCoil). The imaging protocol included bilateral 3D proton-density fat-saturated FSE (ie, Cube) (TR/TE, 1200/27 ms; echo train length, 32; matrix, 512 × 512 pixels; FOV, 16 cm; slice thickness, 0.6 mm [interpolated into 0.3 mm]) for evaluate cartilage and meniscal morphology.

Two radiologists with 9 years and 7 years of experience (V.K. and K.Z.) read the images and graded the MRI findings on the 10-year follow-up images using the modified Whole-Organ Magnetic Resonance Imaging Score (WORMS).^10,11,19,26^ Considering that the original WORMS scale was created for knees with established OA, and our study involved a smaller cohort with expectedly mild degenerative findings, we combined the original 15 compartments into a total of 6: MF, MT, LF, LT, patella, and trochlea. Cartilage and bone marrow lesions were assessed in these 6 compartments by using a modified semiquantitative WORMS, and the highest grade of lesion was recorded for each region.^10,11^ Cartilage signal and morphology were scored with an 8-point scale: 0 = normal thickness and signal, 1 = normal thickness but increased signal, 2 = partial-thickness focal defect <1 cm in greatest width, 2.5 = full-thickness focal defect <1 cm in greatest width, 3 = multiple areas of partial-thickness (grade 2) defects intermixed with areas of normal thickness or a grade 2 defect >1 cm but <75% of the region, 4 = diffuse (at least 75% of the region) partial-thickness loss, 5 = multiple areas of full-thickness loss (grade 2.5) or a grade 2.5 lesion >1 cm but <75% of the region, and 6 = diffuse (at least 75% of the region) full-thickness loss. Subarticular bone marrow abnormalities were defined as poorly marginated areas of increased signal intensity in the normal subchondral and epiphyseal bone marrow. A 4-point grading scale was used to assess the size of the bone marrow abnormalities: 0 = none, 1 = minimal (<25% of region), 2 = moderate (25%-50% of region), and 3 = severe (>50% of region). Meniscal morphology was assessed in 6 regions using a modified WORMS: the anterior, body, and posterior region of the medial and lateral menisci separately. An additional grade was added to the meniscal classification “intrasubstance degeneration” to better assess early degenerative disease. The grading scale ranged from 1 to 4: 0 = normal,1 = intrasubstance abnormalities, 2 = nondisplaced tear, 3 = displaced or complex tear, and 4 = complete destruction. The reconstructed ACL, posterior cruciate ligament (PCL), medial collateral ligament (MCL), and lateral collateral ligament (LCL) were assessed as intact or torn. We calculated the WORMS Total Score as a sum of every point in cartilage, bone marrow lesion, and meniscal gradings, and the WORMS Medial Score as a sum of medial compartment scores in cartilage, bone marrow lesion, and meniscal gradings.

### Statistical Analysis

All statistical analysis was performed using Stata Version 18.5 software (StataCorp). As a preliminary analysis to confirm that the cartilage degeneration characteristics observed in the 13 cases in this study were consistent with our previous findings,^27,28^ paired *t* tests were applied to compare T1ρ/T2 values at baseline and 1 year postoperatively between the ipsilateral and contralateral knees for each cartilage compartment. As primary analyses, the correlations between baseline, 1-year, and delta T1ρ/T2 values and the WORMS Total/Medial Scores at 10 years postoperatively were examined using the Spearman correlation coefficient. As explorational analyses, the correlations between the T1ρ/T2 values in the subcompartments of the MF/MT and the WORMS Total/Medial Scores were also examined using the Spearman correlation coefficient. The significance level was set to .05.

## Results

### Baseline and 1-Year Patient and Knee Data

Patient and knee-specific characteristics are shown in Table 1. The median follow-up period was 124 months (IQR, 121-140 months). The T1ρ and T2 values at baseline and 1 year postoperatively are shown in Table 2. At baseline, the ipsilateral knees had elevated T2 values in MF, LF, and LT (*P* = .023, .0034, and .007, respectively) and elevated T1ρ values in LF and LT (*P* = .011 and .012, respectively) relative to the contralateral knee. One year postoperatively, the ipsilateral knees had elevated T1ρ values in MT (*P* = .0088). The T1ρ values in MF and MT increased significantly from baseline to 1 year (*P* = .0015 and .013) (Table 2), while the T1ρ value in LT decreased significantly (*P* = .026).

**Table 1:** Patient and Surgery Data*^*a*^*

	Value
Sex	
Male	9 (69.2)
Female	4 (30.8)
Age at surgery, y, mean ± SD	32.4 ± 5.4
BMI, mean ± SD	24.2 ± 2.6
Side	
Right	7 (53.8)
Left	6 (46.2)
Time from injury to surgery, days, median (IQR)	42 (38-74)
Graft	
Hamstring tendon autograft	8 (61.5)
Posterior tibialis allograft	5 (38.5)
Medial meniscus	
Intact	11 (84.6)
Partial meniscectomy	2 (15.4)
Repair	00 (0)
Lateral meniscus	
Intact	8 (61.5)
Partial meniscectomy	4 (30.8)
Repair	1 (7.7)
Cartilage	
Intact	11 (84.6)
Medial tibia	
ICRS grade 2	1 (7.7)
Patella	
ICRS grade 3	1 (7.7)
Follow-up period, mo, median (IQR)	124 (121-140)

a Data are expressed as n (%) unless otherwise indicated. BMI, body mass index; ICRS, International Cartilage Regeneration & Joint Preservation Society.

**Table 2:** T1ρ and T2 Values for Each Region at Baseline and 1 Year Postoperatively*^*a*^*

	Relaxation Time, ms		Delta	*P* Value
	Baseline	1 y		
T1ρ				
MF				
Ipsilateral	40.9 ± 2.7	43.2 ± 3.5	2.3 (1.1 to 3.6)	**.0015**
Contralateral	40.3 ± 3.0	41.4 ± 3.6		
Difference	0.6 (–0.60 to 1.9)	1.8 (–0.3 to 3.9)		
*P* value	.29	.089		
MT				
Ipsilateral	34.9 ± 3.0	37.3 ± 4.8	2.4 (0.6 to 4.2)	**.013**
Contralateral	34.6 ± 3.9	34.0 ± 4.3		
Difference	0.3 (–1.4 to 2.1)	3.3 (1.0 to 5.6)		
*P* value	.69	**.0088**		
LF				
Ipsilateral	40.9 ± 1.9	42.3 ± 3.1	1.3 (–0.2 to 2.8)	.075
Contralateral	39.3 ± 2.0	40.5 ± 2.2		
Difference	1.6 (0.4 to 2.8)	1.7 (–0.2 to 3.7)		
*P* value	**.011**	.074		
LT				
Ipsilateral	35.4 ± 2.7	33.4 ± 2.6	–2.0 (–3.7 to −0.3)	**.026**
Contralateral	33.2 ± 1.4	34.6 ± 3.0		
Difference	2.1 (0.6 to 3.7)	–1.2 (–3.3 to 0.9)		
*P* value	**.012**	.24		
T2				
MF				
Ipsilateral	31.8 ± 1.8	32.8 ± 3.2	1.1 (–0.3 to 2.4)	.11
Contralateral	30.7 ± 2.2	32.2 ± 2.6		
Difference	1.1 (0.2 to 2.0)	0.6 (–0.9 to 2.2)		
*P* value	**.023**	.38		
MT				
Ipsilateral	26.6 ± 2.9	27.6 ± 3.0	0.9 (–0.9 to 2.8)	.28
Contralateral	25.5 ± 3.0	26.3 ± 3.3		
Difference	1.1 (–0.2 to 2.5)	1.3 (–0.2 to 2.7)		
*P* value	.090	.090		
LF				
Ipsilateral	31.4 ± 1.6	32.0 ± 2.5	0.6 (–0.5 to 1.8)	.24
Contralateral	30.1 ± 1.4	30.8 ± 1.7		
Difference	1.3 (0.5 to 2.1)	1.2 (–0.2 to 2.6)		
*P* value	**.0034**	.081		
LT				
Ipsilateral	25.2 ± 1.9	24.5 ± 2.4	–0.7 (–2.2 to 0.9)	.36
Contralateral	23.6 ± 1.7	24.4 ± 2.9		
Difference	1.5 (0.5 to 2.5)	0.1 (–1.5 to 1.6)		
*P* value	**.007**	.92		

a Data are expressed as mean ± SD or mean (95% CI) unless otherwise indicated. Delta = T1ρ/T2 values 1 year postoperatively – those at baseline. Difference = ipsilateral knee – contralateral knee. Boldface *P* values indicate a statistically significant difference (*P* < .05). LF, lateral femur; LT, lateral tibia; MF, medial femur; MT, medial tibia.

### Ten-Year Postoperative Outcomes

There were no ligaments assessed as torn in the ACL, PCL, MCL, or LCL on 10-year MRI. The modified WORMS values are shown in Table 3. The median of the 10-year WORMS Total Score was 6.0 points (IQR, 5.0-9.5 points). The median of the 10-year WORMS Medial Score was 1.0 points (IQR, 0-4.0 points).

**Table 3:** WORMS Values at 10-Year Follow-up*^*a*^*

Cartilage		Bone Marrow Lesion		Meniscus	
Medial femur		Medial femur		Medial meniscus	
Grade 0	10 (76.9)	Grade 0	12 (92.3)	Anterior	
Grade 1	00 (0)	Grade 1	1 (7.7)	Grade 0	11 (84.6)
Grade 2	1 (7.7)	Grade ≥2	00 (0)	Grade 1	2 (15.4)
Grade 2.5	2 (15.4)			Grade 2	00 (0)
Grade ≥3	00 (0)			Grade ≥3	00 (0)
Lateral femur		Lateral femur		Body	
Grade 0	11 (84.6)	Grade 0	13 (100)	Grade 0	9 (69.2)
Grade 1	00 (0)	Grade ≥1	00 (0)	Grade 1	00 (0)
Grade 2	00 (0)			Grade 2	3 (23.1)
Grade 2.5	2 (15.4)			Grade 3	1 (7.7)
Grade ≥3	00 (0)			Grade 4	00 (0)
Medial tibia		Medial tibia		Posterior	
Grade 0	12 (92.3)	Grade 0	13 (100)	Grade 0	6 (46.2)
Grade 1	00 (0)	Grade ≥1	00 (0)	Grade 1	2 (15.4)
Grade 2	1 (7.7)			Grade 2	3 (23.1)
Grade 2.5	00 (0)			Grade 3	1 (7.7)
Grade ≥3	00 (0)			Grade 4	1 (7.7)
Lateral tibia		Lateral tibia		Lateral meniscus	
Grade 0	9 (69.2)	Grade 0	11 (84.6)	Anterior	
Grade 1	2 (15.4)	Grade 1	1 (7.7)	Grade 0	10 (76.9)
Grade 2	1 (7.7)	Grade 2	00 (0)	Grade 1	3 (23.1)
Grade 2.5	1 (7.7)	Grade 3	1 (7.7)	Grade ≥2	00 (0)
Grade ≥3	00 (0)			Body	
Patella		Patella		Grade 0	11 (84.6)
Grade 0	6 (46.2)	Grade 0	11 (84.6)	Grade 1	00 (0)
Grade 1	2 (15.4)	Grade 1	00 (0)	Grade 2	2 (15.4)
Grade 2	2 (15.4)	Grade 2	2 (15.4)	Posterior	
Grade 2.5	1 (7.7)	Grade 3	00 (0)	Grade 0	8 (61.5)
Grade 3	2 (15.4)			Grade 1	1 (7.7)
Grade ≥4	00 (0)			Grade 2	4 (30.8)
Trochlea		Trochlea		Grade ≥3	00 (0)
Grade 0	9 (69.2)	Grade 0	13 (100)		
Grade 1	1 (7.7)	Grade ≥1	00 (0)		
Grade 2	1 (7.7)				
Grade 2.5	1 (7.7)				
Grade 3	1 (7.7)				
Grade ≥4	00 (0)				

a Data are expressed as n (%). WORMS, Whole-Organ Magnetic Resonance Imaging Score.

### Correlations Between Baseline, 1-Year, and Delta T1ρ and T2 Values and 10-Year WORMS

**Figure 2.**
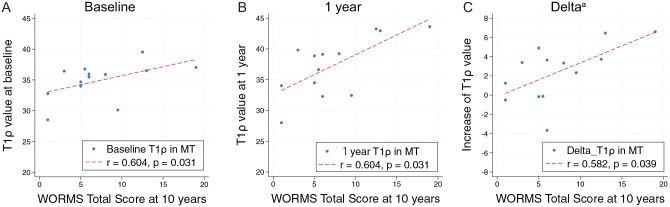
Relationship between T1ρ value and Whole-Organ Magnetic Resonance Imaging Score (WORMS) Total Score at 10 years. (A) T1ρ value in medial tibia (MT) at baseline correlates with 10-year WORMS Total Score. (B) T1ρ value in MT at 1 year correlates with 10-year WORMS Total Score. (C) Delta T1ρ value in MT correlates with 10-year WORMS Total Score. ^a^Delta = T1ρ value at 1 year – T1ρ value at baseline.

The correlations between early cartilage degenerations in MF and MT using qMRI and 10-year WORMS values are shown in Table 4. The baseline, 1-year, and delta MT T1ρ value were significantly associated with the 10-year WORMS Total Score (*r* = 0.604, *P* = .031; *r* = 0.604, *P* = .031; and *r* = 0.582, *P* = .039, respectively) (Figure 2). The T2 value in MT 1 year postoperatively was significantly associated with the 10-year WORMS Total Score (*r* = 0.566, *P* = .046). There was no correlation with the 10-year WORMS Medial Score. The T1ρ and T2 values in LF and those in LT had no associations with the 10-year WORMS Total Score at baseline (*r* = 0.309, *P* = .30; *r* = 0.168, *P* = .58; *r* = −0.019, *P* = .95; and *r* = −0.011, *P* = .97, respectively) and at 1 year (*r* = −0.008, *P* = .98; *r* = 0.055, *P* = .86; *r* = 0.149, *P* = .62; and *r* = −0.058, *P* = .85, respectively). The delta T1ρ/T2 values in LF and those in LT also had no associations (*r* = −0.243, *P* = .42; *r* = 0.075, *P* = .81; *r* = 0.240, *P* = .42; and *r* = −0.221, *P* = .46, respectively).

**Table 4:** Correlations Between Early Cartilage Degenerations Using T1ρ/T2 Values in MF/MT and 10-Year WORMS*^*a*^*

	WORMS Total Score		WORMS Medial Score	
	*r*	*P* Value	*r*	*P* Value
Baseline				
T1ρ				
MF	0.372	.21	0.247	.41
MT	0.604	**.031**	0.483	.095
T2				
MF	0.273	.36	0.073	.81
MT	0.480	.10	0.438	.13
1 y				
T1ρ				
MF	0.130	.67	0.233	.44
MT	0.604	**.031**	0.488	.091
T2				
MF	0.127	.68	–0.042	.89
MT	0.566	**.046**	0.491	.089
Delta				
T1ρ				
MF	–0.121	.69	–0.062	.84
MT	0.582	**.039**	0.474	.10
T2				
MF	–0.039	.9	–0.166	.58
MT	0.232	.44	0.107	.73

a Delta = T1ρ/T2 values 1 year postoperatively – those at baseline. Boldface *P* values indicate a statistically significant correlation (*P* < .05). MF, medial femur; MT, medial tibia; WORMS, Whole-Organ Magnetic Resonance Imaging Score.

For the MF and MT subcompartment analyses, the 1-year and delta T1ρ values in pMT were associated with the 10-year WORMS Total Score (*r* = 0.579, *P* = .040; and *r* = 0.651, *P* = .018, respectively). The delta T1ρ value in pMT was also associated with the 10-year WORMS Medial Score (*r* = 0.668; *P* = .015). The baseline T1ρ value in pMF was associated with the WORMS Total Score (*r* = 0.635; *P* = .022). The other subcompartments were not associated with the 10-year WORMS Total and Medial Scores (Table 5).

**Table 5:** Correlations Between Early Cartilage Degenerations Using T1ρ/T2 Values in Medial Subcompartments and 10-Year WORMS*^*a*^*

	WORMS Total Score		WORMS Medial Score	
	*r*	*P* Value	*r*	*P* Value
Baseline				
T1ρ				
MF				
cMF	0.011	.97	0.233	.44
pMF	0.635	**.022**	0.188	.53
MT				
aMT	0.237	.43	0.393	.18
cMT	0.541	.058	0.474	.10
pMT	0.425	.15	0.132	.66
T2				
MF				
cMF	–0.011	.97	0.286	.34
pMF	0.425	.15	–0.008	.98
MT				
aMT	0.323	.28	0.485	.093
cMT	0.370	.21	0.421	.15
pMT	0.304	.31	0.182	.55
1 y				
T1ρ				
MF				
cMF	–0.022	.94	0.039	.90
pMF	0.041	.89	0.073	.81
MT				
aMT	0.295	.32	0.236	.43
cMT	0.521	.069	0.387	.19
pMT	0.579	**.040**	0.483	.095
T2				
MF				
cMF	0.110	.72	–0.039	.90
pMF	–0.017	.96	–0.213	.48
MT				
aMT	0.367	.21	0.331	.27
cMT	0.406	.17	0.379	.20
pMT	0.513	.074	0.384	.19
Delta				
T1ρ				
MF				
cMF	0.188	.53	–0.149	.62
pMF	–0.483	.095	–0.196	.51
MT				
aMT	–0.063	.83	–0.286	.34
cMT	0.392	.18	0.345	.24
pMT	0.651	**.018**	0.668	**.015**
T2				
MF				
cMF	0.237	.43	–0.185	.540
pMF	–0.439	.13	–0.160	.60
MT				
aMT	<0.001	>.99	–0.373	.21
cMT	0.268	.37	0.084	.78
pMT	0.207	.49	0.084	.78

a Delta = T1ρ/T2 values 1 year postoperatively – those at baseline. Boldface *P* values indicate a statistically significant correlation (*P* < .05). aMT, anterior medial tibia; cMF, central medial femur; cMT, central medial tibia; MF, medial femur; MT, medial tibia; pMF, posterior medial femur; pMT, posterior medial tibia; WORMS, Whole-Organ Magnetic Resonance Imaging Score.

## Discussion

We observed that whole-joint degenerative changes at 10 years after ACLR, as measured by the WORMS Total Score, were significantly associated with increased preoperative and 1-year postoperative T1ρ and T2 values in the MT cartilage. Furthermore, the delta T1ρ values in the MT cartilage, which represent the increase from baseline to 1 year, were also associated with the deterioration of the WORMS Total Score. These results indicate that early cartilage degeneration in the MT detected by qMRI is indeed related to the progression of PTOA after 10 years, which, to our knowledge, is the first such report. This finding holds significance in offering early detection of these degenerative changes that may allow for earlier noninvasive evaluation of the success of interventional treatment strategies in preventing PTOA after ACLR.

Previous studies conducted on early postoperative qMRI evaluations after ACLR mainly reported increased T1ρ and T2 values in the cartilage of the medial femorotibial compartment.^1,6,9,16,27,30,31^ Our past reports, which include cases that formed the basis of this study cohort,^1,27,30^ had similar results. Those reports noted that early postoperatively, T1ρ values in the MT cartilage of the injured knee are increased compared with the uninjured knee.^1,27^ Theologis et al^30^ reported that the presence or absence of a meniscal tear increases the risk of degeneration in the femorotibial cartilage. In another large multicenter prospective study, it has been confirmed that the extension of T1ρ/T2 values in the medial and lateral femoral condyles and degeneration in the uninjured knee have also been reported.^6^ Numerous studies have used T1ρ/T2 imaging to evaluate cartilage degeneration; however, to our knowledge, no existing reports have explored whether this detected cartilage degeneration correlates with actual PTOA progression 10 years later. The current study demonstrates the important correlations between the early cartilage degeneration in MT and the WORMS Total Score 10 years postoperatively. These findings substantiate the ability and significance of detecting early cartilage degeneration through qMRI.

The elevated qMRI values before ACLR in the LT region were reported in the previous studies.^5,16,27,34^ In the prospective cohort NACOX study, the increases in preoperative values in the medial compartment compared with the healthy side were reported.^5^ In our review, we found only 1 report addressing the correlation between preoperative values and long-term PTOA. Watanabe et al^34^ reported that the preoperative T1ρ value in the posterior LT was associated with the radiographic progression of PTOA, and they also noted that cases with preoperative OA changes tended to progress faster postoperatively. We suggest in the current study that the preoperative T1ρ value in MT is associated with the 10-year WORMS Total Score, which may imply the detection of cases already exhibiting OA changes, which is a similar result to the previous literature.^34^ However, the 1-year T1ρ value and the increase in T1ρ values from preoperatively to 1 year postoperatively are also related to the WORMS Total Score, indicating that both the preoperative increase and the subsequent rise are linked to outcomes 10 years later.

The primary significance of the current study lies in its demonstration of a connection between early cartilage degeneration and the actual progression of PTOA. Considering that early cartilage degeneration, whether postinjury or postoperative, is indeed related to PTOA progression 10 years later, this finding suggests its potential use as a predictive tool for early postsurgical PTOA progression. However, the direct linkage of these changes to subsequent OA progression remains uncertain. This study highlights this relationship, potentially affirming its utility as a future diagnostic tool.

Our results suggested that the T1ρ value was potentially better suited for capturing subtle biochemical abnormalities in the early stages than the T2 value. T1ρ and T2 quantification are known methods for noninvasively evaluating changes in the biochemical composition of articular cartilage, and their values generally increase with cartilage degeneration.^15,20,32^ However, the 2 reflect different relaxation mechanisms, and there is a potential difference in their sensitivity for detecting early degeneration. Previous research^14,22^ has shown that elevations in T1ρ value indicate proteoglycan abnormalities and increases in T2 signal disarray in collagen fibers. Multiple studies have reported that the T1ρ value has higher sensitivity for detecting early cartilage degeneration compared with the T2 value.^17,20,22,29^ Furthermore, the spin-lock pulse of the T1ρ value has the advantage of reducing the influence of dipolar interactions due to collagen fiber orientation and the magic angle effect, to which the T2 value is susceptible.^32^ T1ρ is particularly expected to be useful for prediction in the early stages, because of its ability to detect initial proteoglycan changes and its reduced magic angle effect. The results of this study are also considered to support this characteristic of the T1ρ value.

### Limitations

This study has some limitations. First, the size of our cohort is small and represents only a subset of the previous cohort. Comparisons between injured and uninjured sides used data from the uninjured side as the control, but overall, there may have been insufficient statistical power. However, because this cohort underwent qMRI and was followed for 10 years after surgery, it is extremely valuable in supporting the high fidelity of the data. Second, the analysis was limited to univariate methods, and it did not account for confounding factors such as age. It is likely that factors such as meniscal status and age influence the postoperative outcomes after ACLR. Therefore, to analyze these effects in detail, it would be necessary to increase the number of cases and use multivariate analysis; however, as previously mentioned, securing a sufficient number of cases remains challenging and is an issue for future research. Nevertheless, the finding of a relationship between qMRI and outcomes 10 years later is in itself a significant contribution of this study. Third, it should be noted that the TE values used in the MRI protocol for calculating T2 values differed slightly between groups of 8 and 5 patients. However, because the same MRI scanner and coils were used, and the differences in TE were minimal, with the same number of imaging shots (n = 4) used for calculating T2 values, it is reasonable to believe that these differences had a negligible effect on the T2 values and did not affect the results. Fourth, our study did not include a detailed analysis of graft selection during surgery or concomitant injuries such as meniscal tears. Finally, the evaluation at 10 years after surgery is based on the WORMS, which does not capture quantitative changes in cartilage. However, for the purpose of this study, we believe it is more appropriate to examine morphological changes rather than qMRI values at 10 years. The WORMS is widely known as a method that comprehensively captures various OA changes. Therefore, it excels not only in scoring cartilage degenerative findings but also in scoring overall knee OA progression, making it highly suitable for measuring the progression of PTOA in the entire knee.

## Conclusion

The T1ρ/T2 values 1 year postoperatively and the increase from baseline to 1 year postoperatively in the MT cartilage after ACLR were associated with knee joint deterioration measured 10 years postoperatively. These findings help establish qMRI as a valuable biomarker for detecting early signs of PTOA progression.
